# Newly Synthesized Doxorubicin Complexes with Selected Metals—Synthesis, Structure and Anti-Breast Cancer Activity

**DOI:** 10.3390/molecules22071106

**Published:** 2017-07-04

**Authors:** Agata Jabłońska-Trypuć, Grzegorz Świderski, Rafał Krętowski, Włodzimierz Lewandowski

**Affiliations:** 1 Division of Sanitary Biology and Biotechnology, Faculty of Civil Engineering and Environmental Engineering, Białystok University of Technology, Wiejska 45E Street, Białystok 15-351, Poland; 2 Division of Chemistry, Bialystok University of Technology, Białystok 15-351, Poland; g.swiderski@pb.edu.pl (G.Ś.); w.lewandowski@pb.edu.pl (W.L.); 3 Department of Pharmaceutical Biochemistry, Medical University of Białystok, Białystok 15-222, Poland; r.kretowski@umb.edu.pl

**Keywords:** doxorubicin, metals, breast cancer, MCF-7

## Abstract

Doxorubicin (DOX) is very effective chemotherapeutic agent, however it has several major drawbacks. Therefore the motivation for developing novel drug complexes as anticancer agents with different mechanism of action has arisen. The aim of the present study was to evaluate the influence of newly synthesized DOX complexes with selected metals (Mg, Mn, Co, Ni, Fe, Cu, Zn) on apoptosis, cell cycle, viability, proliferation and cytotoxicity in the breast cancer cell line MCF-7. Complexation of DOX with metals has likewise been the subject of our research. The current work showed that the tested bivalent metals at a given pH condition formed metal:DOX complexes in a ratio of 2:1, while iron complexes with DOX in a ratio of 3:1. The studies also showed that selected metal-DOX complexes (Mg-DOX, Mn-DOX, Ni-DOX) at 0.5 µM concentration significantly decreased cell viability and proliferation, however they increased caspase 7 activity. Results also indicated that studied metal-DOX complexes showed high cytotoxicity in MCF-7 cells. Therefore they were chosen for cell cycle check-points and apoptosis/necrosis analysis studied by flow cytometry. Obtained results suggest that doxorubicin complexed by specified metals can be considered as a potential anti-breast cancer agent, which is characterized by a higher efficacy than a parent drug.

## 1. Introduction

The most commonly diagnosed cancers worldwide are lung cancer, breast cancer and colorectal cancer, however, in women, breast cancer is the most common malignant tumor. This is the most frequently occurring cancer among women especially in developed countries. Every year in the UK it is diagnosed in over 44,000 women and the incidence has increased over the past 20 years by 50 percent [1].

**Figure 1:**
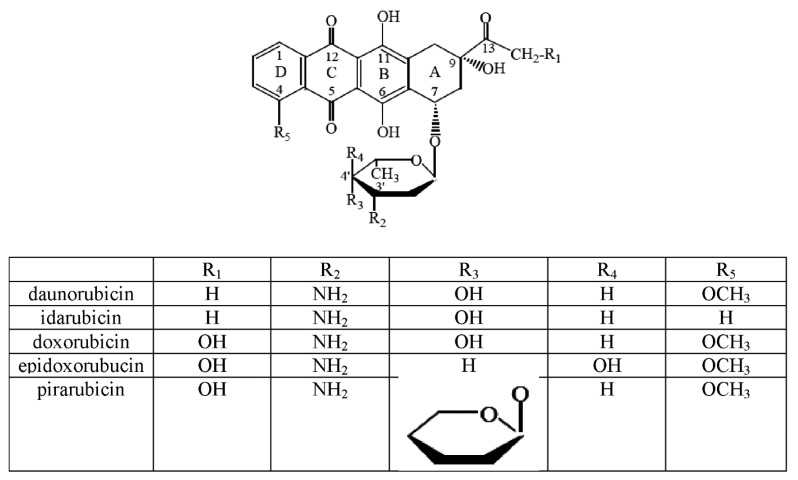
Structural formula of the selected anthracyclines regarding the functional groups of the individual compounds.

Breast cancer is characterized by following features: frequent aggressive invasion, early metastasis and resistance to multiple drugs used in therapy. All the above mentioned factors suggest the need for theexploration of the novel, more efficient anticancer agents without disruptive side effects. Doxorubicin (DOX) belongs to the anthracycline group—a class of drugs that are commonly used for breast cancer chemotherapy, often in conjunction with other compounds [2]. In the chemical structure of anthracyclines two parts can be distinguished: the aglycone, that consists of four rings two of which are aromatic rings (B and D), and the saccharide moiety (Figure 1). Rings indicated by symbols A, B, C and D differ from each other. Ring B with the hydroquinone structure differs from the C ring, which is devoid of hydroxyl groups. The side chain is located at the C-9 position of the A ring. It is linked to the carbonyl group and at the C-7 position of the amino sugar (daunosamine) and it is attached by a glycosidic linkage. Ring D has in turn a methoxyl group in the C-4 position. In the anthracycline antibiotic molecule several asymmetric carbon atoms can be distinguished: two of them in the aglycone (C-7 and C-9) and four in the sugar moiety (C-1, C-3, C-4, C-5). The presence of a carboxyl group (ring A), hydroxyl group (ring B) and the sugar moiety affect the interactions of the macromolecules within cells [3].

The above described chemical structure is characteristic for the anthracycline antibiotics belonging to the class I, such as DOX, daunorubicin, epirubicin, idarubicin, pirarubicin [4]. Anthracyclines contain in their chemical structure a fragment of both hydrophilic and hydrophobic properties, which allows them to binding to the plasma proteins and cellular membranes. Due to their polar nature they dissolve in water and display both acidic and basic properties. These compounds have crystalline structures and are stable at room temperature [5].

They are known to have cytotoxic properties against tumor cells through three different mechanisms. The first one is the intercalation between strands of DNA/RNA molecules, which results in interference with DNA/RNA synthesis in rapidly dividing cells, such as breast cancer cells [6]. The second mechanism is the inhibition of topoisomerase II activity, which is based on the binding of the anthracycline with the DNA-topoisomerase complex. Anthracyclines, such as doxorubicin, belong to the group of topoisomerase II poisons and therefore they may immobilize topoisomerase II-DNA complexes. For this reason they cause an inhibition of the release of DNA breaks generated by the enzyme. According to the literature, “catalytic inhibitors” inhibit enzymes binding to DNA substrate or cause the closing of the topoisomerase II in the form of a bracelet that surrounds DNA at the post-religation step. However, only topoisomerase II poisons activity can produce DNA breaks [7]. The third mechanism by which selected compounds from the anthracycline group influence cancer cells metabolism is the creation of iron-mediated oxygen free radicals [8].

Literature data indicate that the pharmacological properties of anthracyclines are indirectly connected with the presence of metal ions and with the environment of the metal-antibiotic chelates [9,10]. The investigations associated with metals such as Cu(II), Hg(II), Ag(I), Ni(II) and Mg(II) binding to anthracyclines have shown that the metal has the possibility to bound both to aglycone ring and to the sugar moiety—daunosamine. Furthermore, a considerable limitation is the solubility of the drug. Close to the solubility ratio metal-anthracyclines complexes have the capacity to create dimers [11]. The complexation of the anthracyclines by the metal ions led to discovery of the new, less toxic anticancer agents. The structure of the complexes, the impact that they have on redox reactions and the characteristics of the selected metal binding is still under investigation [12,13,14]. The locations where the metal is bound to the anthracycline are aglycone inner rings depending on the metal nature, with the simultaneous consideration of the ligand spatial structure (Table 1).

Metal-DOX complexes, by the increase of the oxidative stress level, could potentially induce apoptosis. Fe^3+^-DOX complex bound up with DNA, is stable in aqueous solution and in addition it can be reduced to Fe^2+^ through the action of reducing agents such as NADPH dependent cytochrome P_450_ reductase, glutathione or cysteine. These reactions are accompanied by the creation of superoxide anion and the conversion of anthracycline quinone to semiquinone free radicals. During the Haber-Weiss reaction, which is catalyzed by iron ions, hydrogen peroxide and highly reactive hydroxyl radicals are produced. Semiquinone radicals have the ability to transform into a C7 aglycone radical, which is a potent alkylating agent. Reactive oxygen species created with the participation of anthracyclines, cause DNA damage and lead to the apoptosis [20,21].

Apoptosis is a kind of programmed cell death, that occurs in multicellular organisms and it is important for homeostasis, normal cells and tissues growth and development, but also for cancer treatment. Any changes in the process of apoptosis significantly influence cells and tissues metabolism, e.g., may cause an abnormal cell growth, uncontrolled cell divisions and generation of mutations. Consequently, the control and regulation of apoptosis is a main target for new anticancer therapies [22,23]. In mammalian cells exist at least two pathways, by which an apoptosis occurs: an extrinsic death-receptor dependent apoptosis and intrinsic mitochondrial dependent apoptosis. In both above mentioned pathways the induction of cell death is connected with selected caspases activation: initiator caspases (e.g., caspase-8 and -9) and effector caspases (e.g., caspase-3, -6, and -7).

The purpose of this study was to evaluate the influence of selected, newly synthesized metal-DOX complexes on breast cancer cells proliferation, viability, cytotoxicity and apoptosis. The molar ratio of created metal-DOX complexes was determined by spectrophotometric titration in buffer solution at pH = 7.00. Next, FT-IR and FT-Raman spectra of doxorubicin and UV-Vis spectra of metal—doxorubicin complexes were taken. Because DOX and other anthracyclines cause severe side effects, a need to synthesize new, more efficient DOX complexes with selected metals arises.

The current work is a part of the project involving several different aspects. The first one is the evaluation of antitumor activity of metal complexes as compared to selected ligands. The second are relational studies regarding molecular structure and distribution of electrical charge and cytotoxic effects of studied compounds. The third aspect is the study of synergistic effects, in other words mutual reinforcement of antineoplastic action of metal and ligand (with proven cytotoxic activity). The influence of metals on the distribution of electronic charge (determining biological properties) of selected ligands has been described in our earlier articles [24,25,26,27].

## 2. Results

### 2.1. IR and Raman Spectra of DOX

**Figure 2:**
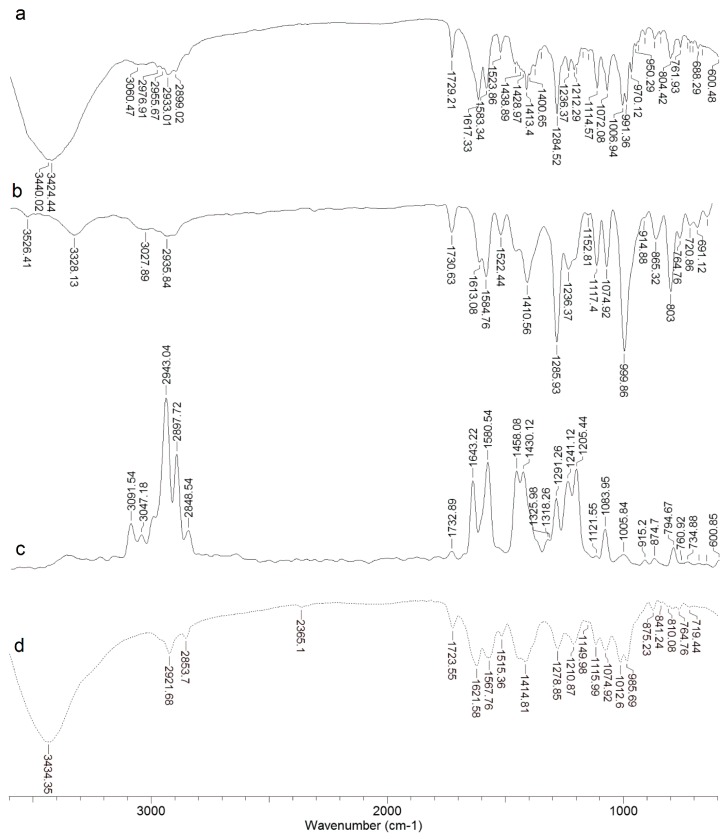
IR KBr (**a**) and ATR (**b**) and Raman (**c**) spectra of DOX (continuous lines) and IR KBr spectra of Cu-DOX complex (the dotted line) (**d**).

In the infrared and Raman spectrum DOX is characterized by some characteristic bands. At 3423 cm^−1^ we observed a wide, strong band coming from the stretching vibration of the hydroxyl group vH-OH, which demonstrates that the substance was hydrated. This band can mask other bands in the 3500–2900 range that are visible in the Raman spectrum (3364, 3092 cm^−1^). The Raman spectra (in the range of 3500–2850 cm^−1^) have intense bands derived from the C-H chain tension of the sugar ring. In the IR spectrum these vibrations are low in intensity. The oscillation of the aromatic ring occurs throughout the spectral range. C-H oscillations derived from aliphatic groups (e.g., methoxy groups). At a wavelength of about 1730 cm^−1^, a characteristic midrange band from the δN-H bending oscillation of the amino group of the sugar ring appears in the IR and Raman spectra. In the IR and Raman spectra at a wavelength of about 1007 cm^−1^ a midrange intensity band is generated from the oscillation of the C-O-C glycosidic moiety (Figure 2, Table 2).

### 2.2. Study on the Composition of Metal-DOX Complexes in Aqueous Solutions and Solid Phase

**Figure 3:**
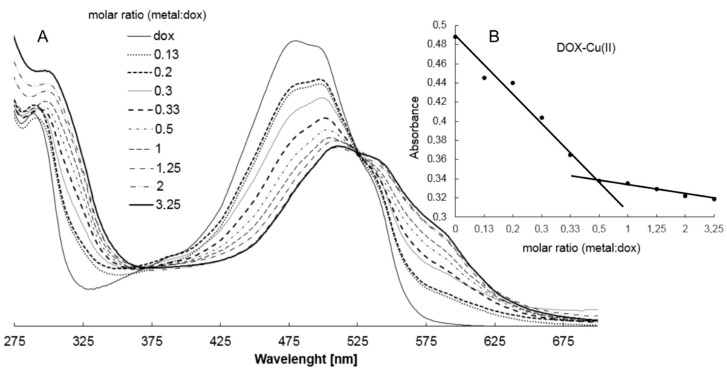
(**A**) Absorption spectra of DOX in the presence of various amounts of Cu(II) in Tris-HCl buffer pH 7.01 (different types of lines are labeled with a molar ratio Cu(II):DOX 0.13–2.0); (**B**) absorbance as a function of [Cu(II)]:[DOX] molar ratio at 500 nm.

**Figure 4:**
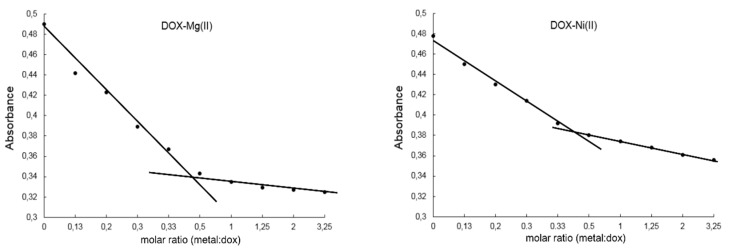

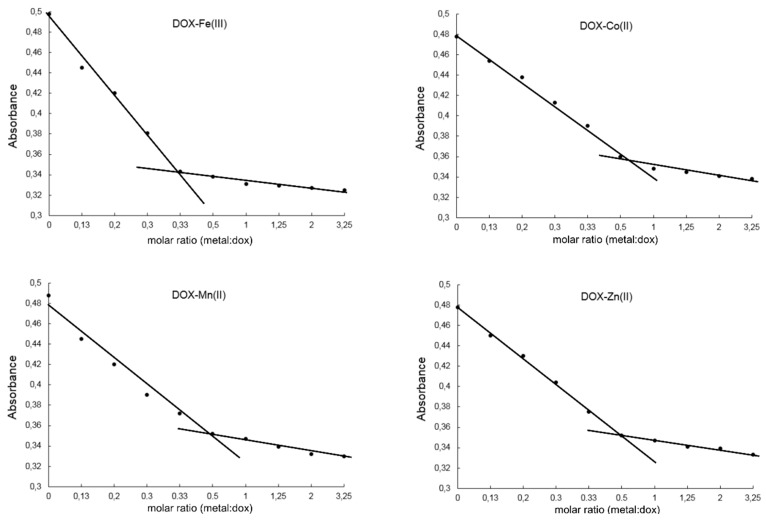
Absorbance as a function of [Metal]:[DOX] molar ratio at 500 nm. Metal-Dox complexes are water-soluble and they were prepared in Tris-HCl buffer pH 7.01.

**Figure 5:**
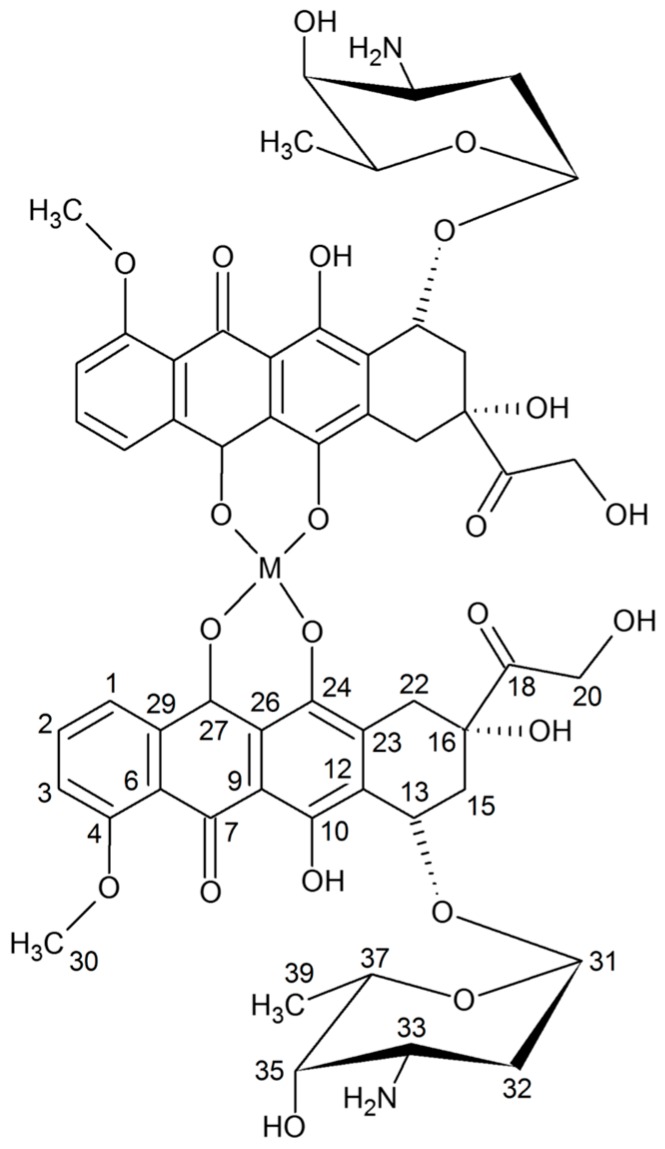
Proposal structure of complex metal-DOX.

The composition of DOX complexes with metals such as Fe(III), Cu(II), Mn(II), Ni(II), Co(II), Mg(II), Zn(II) was investigated. The complex composition in aqueous solution was tested in the presence of the buffer (Tris-HCl) at pH 7.01. The results of the study on the composition of the complexes are depicted in Figure 3 and Figure 4. Figure 3A shows the UV-VIS spectra of DOX and DOX complexes with copper, and in Figure 3B the changes in the maximal absorption of DOX by copper complexation are presented. An analytical band of 510 nm wavelength was selected. The other presented curves reflect the measurements carried out for the DOX solution after the addition of stoichiometric amounts of copper(II) chloride at a concentration of 0.01 M. Changes in the maximum absorbance of the resulting copper complex are shown in Figure 3. The maximum absorbance after addition of copper ions decreases sharply to 0.34. The value is reached for a molar ratio of 1:2 (copper: DOX) and then falls slowly. As the amount of copper ions increases, the absorbance value of DOX decreases as a result of complexation. In the other solutions, where the amount of metal ions is higher than the metal: DOX 1:2 molar ratio, the absorbance decreases slightly. On the basis of the graphs of the absorbance ratios of the metal:DOX molar ratio it was found that copper forms a complex with DOX in a ratio of 1:2 (metal:DOX). Analogous measurements were conducted for DOX complexed with nickel, zinc, magnesium, cobalt, iron and manganese. Changes in the maximum of absorbance of DOX complexed with the investigated metals are shown in Figure 4. Based on the literature data and IR spectroscopy results the probable structures of the complexes were determined (Figure 5) [13,15,16,17,18,19,28]. In the IR spectra of the complexes, the bands associated with the oscillation of the hydroxyl group and the carbonyl group (e.g., bands at 970 cm^−1^ and 912 cm^−1^ in the doxorubicin spectrum) have been lost. The spectra of the selected complex (Cu-DOX) are shown in Figure 2. The spectral data are summarized in Table 2 In each of the analyzed complexes, the metal is attached to the DOX molecule by substituting the hydrogen atom of the hydroxyl group at C (24) and by the attaching to the oxygen atom of the carbonyl group C (27) (Figure 5). In the case of the Fe(III)-DOX complex, the metal coordinates three ligands, however the metal—ligand coordination mode is similar like in other studied metal-anthracyclin complexes (through the carbonyl group and the hydroxyl group).

### 2.3. Estimation of Cells Proliferation

**Figure 6:**
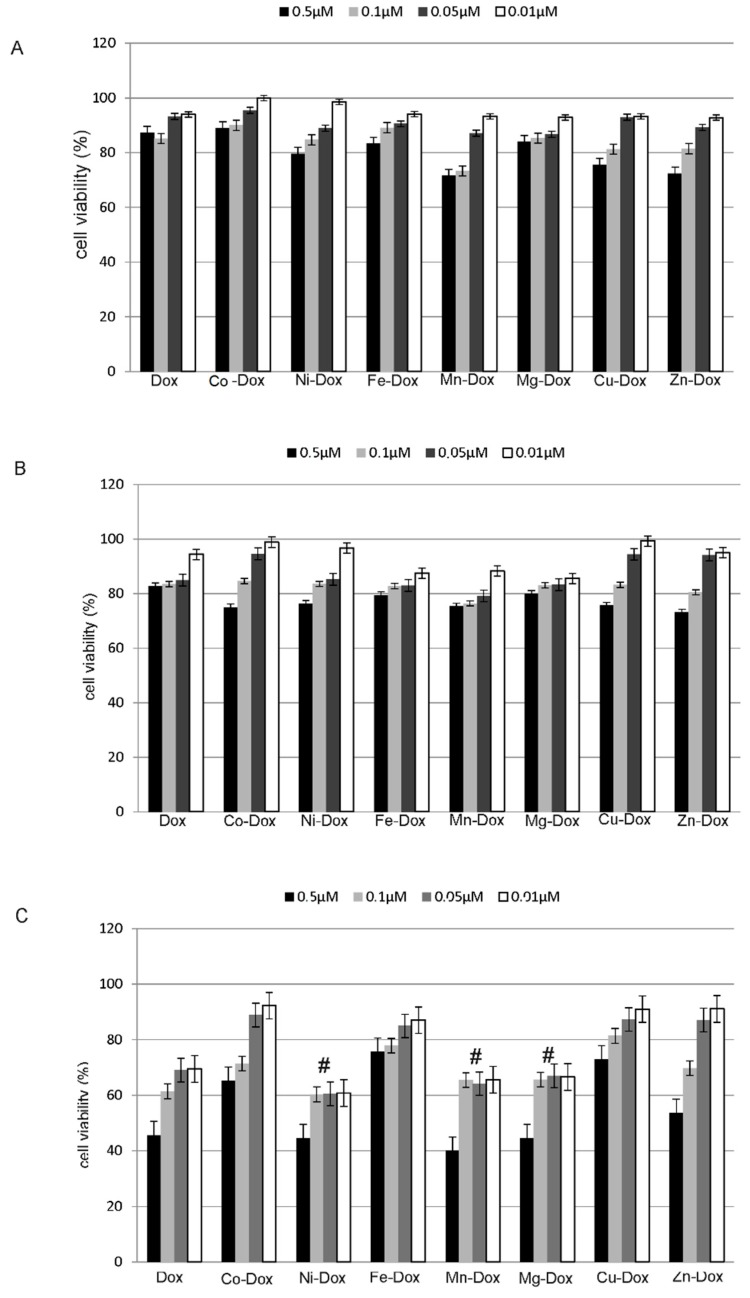
The effect of DOX, Cu-DOX, Zn-DOX, Co-DOX, Ni-DOX, Fe-DOX, Mn-DOX and Mg-DOX on cell proliferation of MCF-7 cells. The cells were incubated with 0.5 µM, 0.1 µM, 0.05 µM and 0.01 µM DOX and Metal-DOX complexes for 2 h (**a**), 4 h (**b**) and 24 h (**c**). Mean values from five independent experiments ± SD are shown. Significant alterations are expressed relative to DOX—controls and marked with crosses (#). Statistical significance was considered if # *p* < 0.05.

In the cell proliferation assay, which is based on the integrity of cell membranes and it was conduct by using the Luna Logos Biosystem cell counter and trypan blue dye, a significant decrease in MCF-7 cancer cells proliferation was observed both as a result of incubation with doxorubicin and its complexes with selected metals. The most significant decreases in cells proliferation were observed in case of Mg-DOX, Mn-DOX and Ni-DOX in 0.5 µM concentration after 24 h treatment. After 2 h and 4 h treatment, especially in lower concentrations of tested compounds no significant decreases in cells proliferation were observed. The influence of selected metals on cells proliferation after 2 h, 4 h and 24 h treatment is depicted in Figure 6.

### 2.4. Estimation of Metals Cytotoxicity

**Figure 7:**
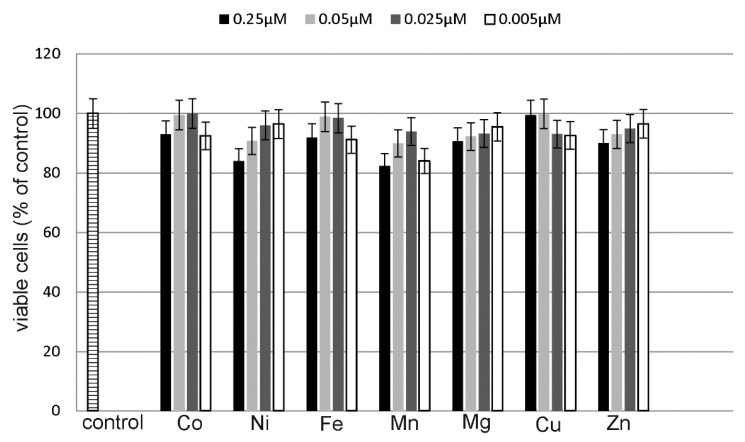
Co, Ni, Fe, Mn, Mg, Cu and Zn cytotoxicity in MCF-7 cells. The cells were incubated with 0.25 µM, 0.05 µM, 0.025 µM and 0.0005 µM of metal chlorides for 24 h. Mean values from five independent experiments ± SD are shown. Results are statistically insignificant.

A CytoTox-Glo Cytotoxicity Assay (Promega) was used to assess the cytotoxic effects of the studied metal salts on MCF-7 cell line. The studied cells were exposed to different concentrations of CuCl_2_, ZnCl_2_, CoCl_2_, NiCl_2_, FeCl_3_, MnCl_2_ and MgCl_2_ for 24 h. The tested concentrations were twice as low as the metal-DOX complexes concentrations. None of the tested compounds showed statistically significant increase in cytotoxicity (Figure 7).

### 2.5. Estimation of Cells Viability, Cytotoxicity and Apoptosis

**Figure 8:**
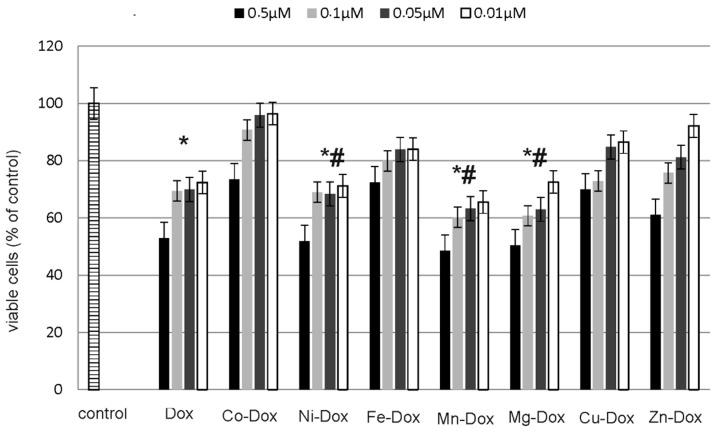
The effect of DOX, Cu-DOX, Zn-DOX, Co-DOX, Ni-DOX, Fe-DOX, Mn-DOX and Mg-DOX on cell viability of MCF-7 cells. The cells were incubated with 0.5 µM, 0.1 µM, 0.05 µM and 0.01 µM DOX and Metal-DOX complexes for 24 h. Mean values from five independent experiments ± SD are shown. Significant alterations are expressed relative to control untreated cells (marked with asterisks *) and to DOX—controls (marked with crosses #). Statistical significance was considered if *# *p* < 0.05.

**Figure 9:**
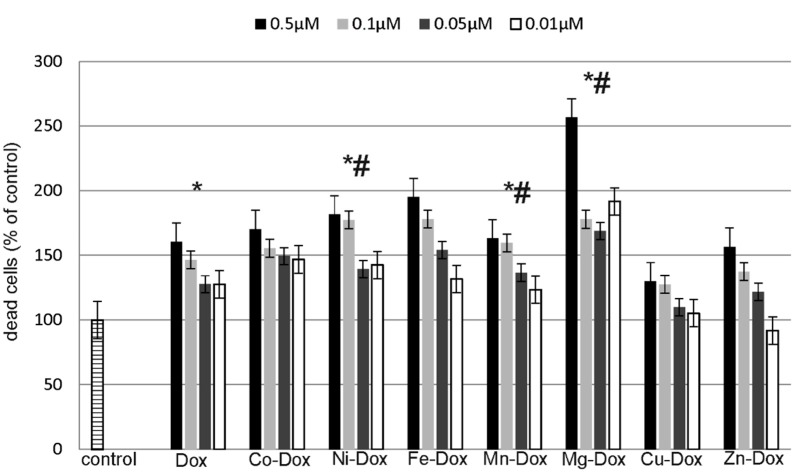
DOX, Cu-DOX, Zn-DOX, Co-DOX, Ni-DOX, Fe-DOX, Mn-DOX and Mg-DOX cytotoxicity in MCF-7 cells. The cells were incubated with 0.5 µM, 0.1 µM, 0.05 µM and 0.01 µM DOX and Metal-DOX complexes for 24 h. Mean values from five independent experiments ± SD are shown. Significant alterations are expressed relative to control untreated cells (marked with asterisks *) and to DOX—controls (marked with crosses #). Statistical significance was considered if #* *p* < 0.05.

**Figure 10:**
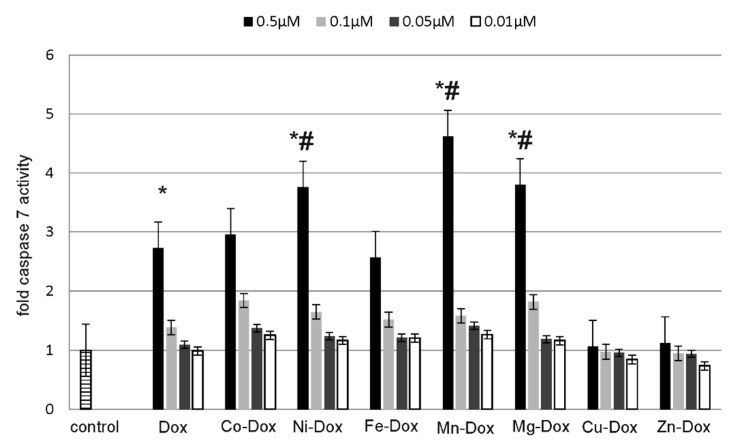
The effect of DOX, Cu-DOX, Zn-DOX, Co-DOX, Ni-DOX, Fe-DOX, Mn-DOX and Mg-DOX on apoptosis in MCF-7 cells. The cells were incubated with 0.5 µM, 0.1 µM, 0.05 µM and 0.01 µM DOX and Metal-DOX complexes for 24 h. Mean values from five independent experiments ± SD are shown. Significant alterations are expressed relative to control untreated cells (marked with asterisks *) and to DOX—controls (marked with crosses #). Statistical significance was considered if *# *p* < 0.05.

ApoTox-Glo™ Triplex Assay (Promega) was used to assess the apoptosis and cytotoxic effects of DOX and its metal complexes on MCF-7 cell line. The studied cells were exposed to different concentrations of DOX and Cu-DOX, Zn-DOX, Co-DOX, Ni-DOX, Fe-DOX, Mn-DOX and Mg-DOX for 24 h. All of the tested compounds caused dose-dependent reduction in cells viability as compared to the control, non-treated cells (Figure 8). Particularly it was observed in case of 0.5 µM concentration of Mg-DOX, Mn-DOX and Ni-DOX. Simultaneously, a significant increase in cytotoxicity of Mg-DOX, Mn-DOX and Ni-DOX (0.5 µM) was noticed (Figure 9). Likewise, an increased level of apoptosis was observed in the presence of especially Mg-DOX, Mn-DOX and Ni-DOX (0.5 µM). Exposure of MCF-7 cells to those three compounds significantly increased caspase 7 activity and thus apoptosis (Figure 10).

### 2.6. Detection of Apoptosis and Necrosis

**Figure 11:**
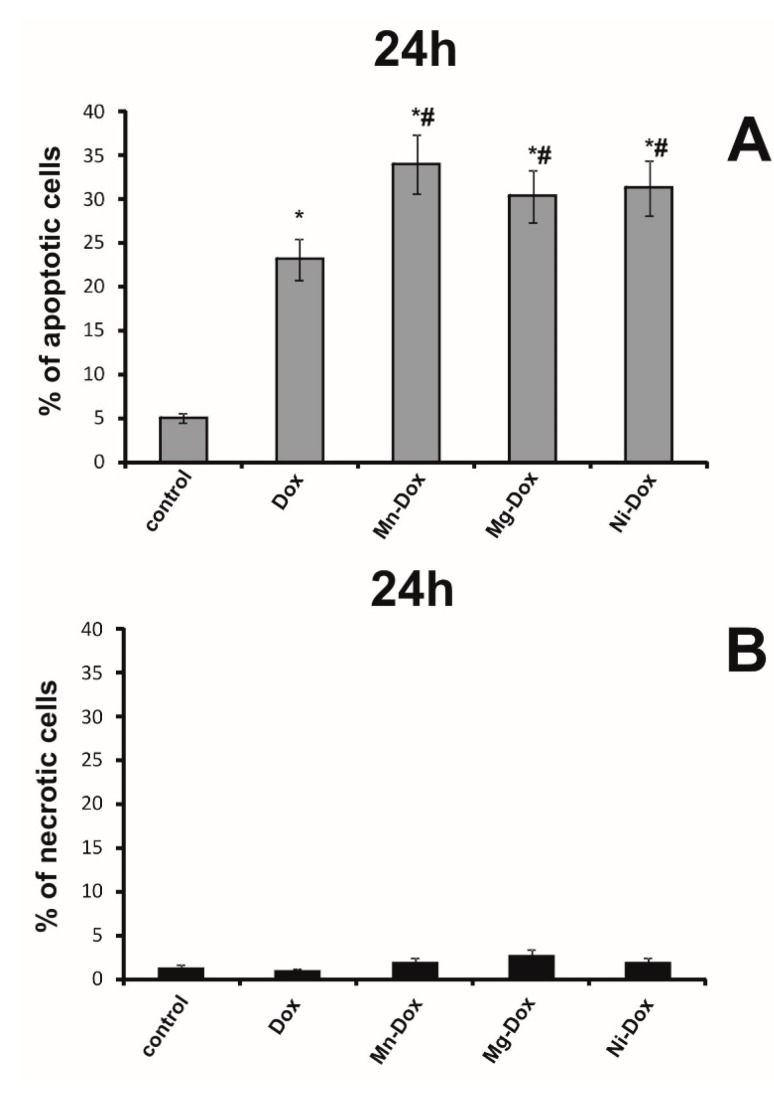
The effect of DOX, Ni-DOX, Mn-DOX and Mg-Dox on apoptosis of MCF-7 cells. The cells were incubated with 0.5 μM DOX, Ni-DOX, Mn-DOX and Mg-DOX for 24 h. Bar graphs presenting the percentage of apoptotic MCF-7 (**A**) and necrotic MCF-7 (**B**) cells, are demonstrated. Mean values from five independent experiments ± SD are shown. Significant alterations are expressed relative to control and marked with asterisks (*); significant alterations are expressed relative to DOX-control and marked with crosses (#). Statistical significance was considered if *# *p* < 0.05*.*

An apoptosis in MCF-7 cells was estimated by using flow cytometry on FACSCanto II cytometer (Becton-Dickinson, San Diego, CA, USA). Figure 11 shows the percent of apoptotic and necrotic cells in cultures incubated for 24 h with DOX, Mg-DOX, Mn-DOX and Ni-DOX in 0.5 µM concentration. We observed significant changes between the tested complexes. The concentration of 0.5 µM Mn-DOX resulted in the most significant increase in apoptosis as compared to DOX-treated cells. We didn’t observed significant changes between the tested metal complexes.

### 2.7. Cell Cycle Analysis

**Figure 12:**
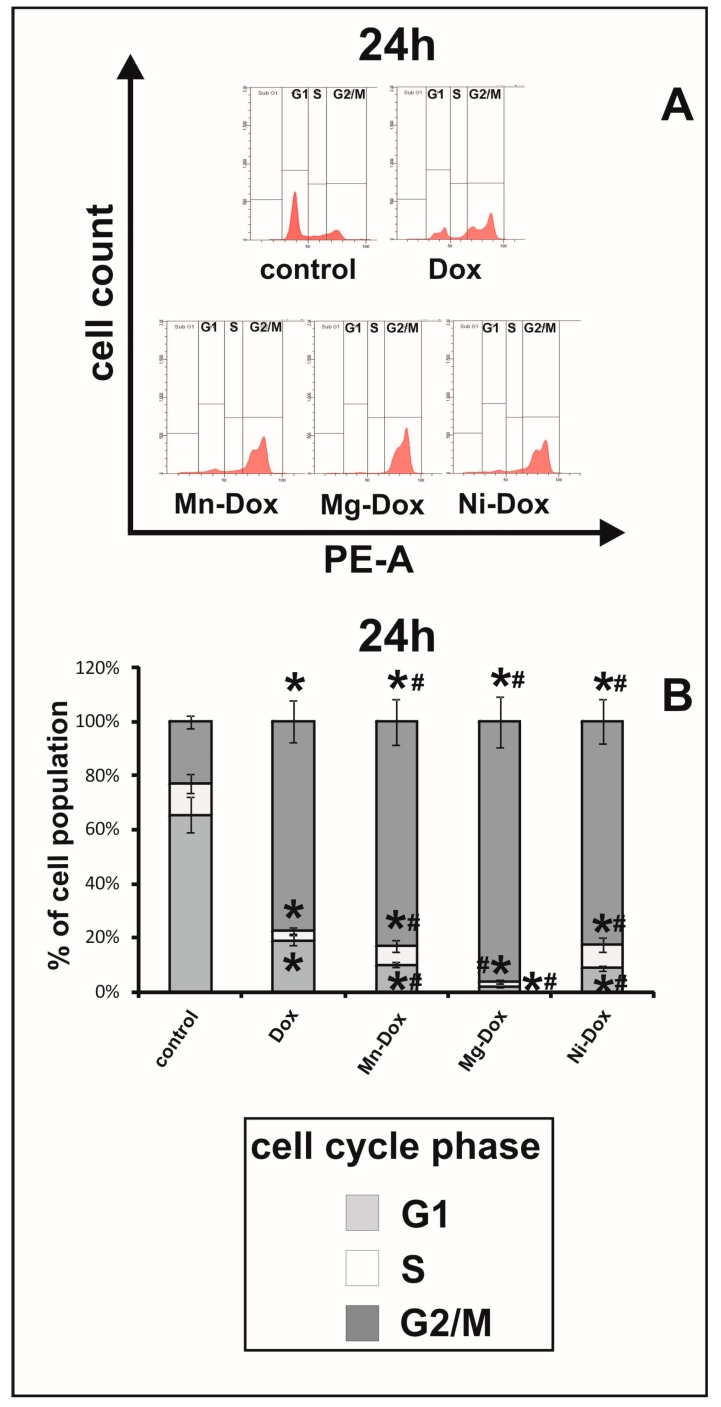
The effect of DOX, Ni-DOX, Mn-DOX and Mg-DOX on cell cycle distribution of MCF-7 cell line. The cell cycle was measured by propidium iodide staining followed by flow cytometry analysis. Results are shown for cells treated with 0.5 μM Ni-DOX, Mn-DOX and Mg-DOX for 24 h versus untreated controls and versus 0.5 µM DOX. Graphical representation of the cell cycle profiles obtained from flow cytometry measurements in MCF-7 cells is depicted in (**A**). Bar graph presenting the percentage of cell cycle distribution in MCF-7 after 24 h (**B**) of treatment with DOX, Ni-DOX, Mn-DOX and Mg-DOX. Significant alterations are expressed relative to control and marked with asterisks (*); significant alterations are expressed relative to DOX-control and marked with crosses (#). Statistical significance was considered if *# *p* < 0.05.

To investigate the connection between anti-proliferative activity of DOX and Ni-DOX, Mn-DOX and Mg-DOX (0.5 µM, 24 h) and cell-cycle arrest, flow cytometry was used. Twenty four hour treatment with DOX-metal complexes: Mg-DOX, Mn-DOX and Ni-DOX in 0.5 µM concentration, affected the cell cycle in studied cell line (Figure 12). A significant increase in the number of cells in G2/M phase with a simultaneous decrease in S and G1 phase was observed in all of the tested compounds as compared to DOX. The results were also referred to the control, non-treated cells. Analysis of flow cytometry results revealed also that MCF-7 cells cultured with DOX and its metal complexes showed significant differences in the distribution of cell cycle phases, with more pronounced effects observed in case of Mg-DOX than the other tested compounds.

## 3. Discussion

The success of cisplatin has led to increased interest in other metal complexes as potential anticancer agents. Especially metals, such as zinc, copper, iron, manganese and others, which are involved in variety of biological processes, are increasingly applied to treating multiple disorders including different types of cancer [29,30]. One of the very important metal features is the fact that in aqueous solutions they have the ability to form positively charged ions that might bind to negatively charged molecules. Therefore the charge can be changed depending on the coordination environment, which leads to the generation of cationic, anionic or neutral species. Moreover, metal ions that are characterized by high electron affinity can polarize groups coordinated to them, promoting hydrolysis reactions [31]. Compounds that contain metals in their structure have many advantages in contrast to traditional carbon-based agents. These benefits mainly due to the fact that they have the ability to coordinate ligands in a three dimensional structure and therefore the functional groups can be adapted to the molecular targets. Complexes, in which metals are involved, can create an environment that favors the building of many different molecular structures that endow a wide range of coordination numbers and geometries and kinetic properties [32,33,34].

Complexation of DOX with metals has been the subject of numerous studies described in the literature. Feng et al. studied DOX complexes with copper(II) and iron(II) [9]. Spectroscopy (UV-Vis) and spectrofluorometry showed that DOX was complexed with copper in a ratio of 2:1 and with an iron in a ratio of 3:1. DOX was complexed with an iron and copper ions at pH = 7 in Tris-HCl buffer. According to the literature metal-DOX complexes exhibit different stability, e.g., doxorubicin complex with iron(III) shows higher stability than the copper complex [9,18]. Studies by Benny et al. have demonstrated that DOX complexed with manganese at pH 7 (HEPES buffer) forms a ligand:metal complex in a 2:1 ratio [28]. The formation of Mn(II)-doxorubicin complexes, with a high stability constant (p*K_d_* = 7.0) has been also described [28]. Literature data show that the complexation of doxorubicin with metal ions can increase the cytotoxic properties of the drug and enhance the accumulation of drug in the cells. Available literature data indicate that much attention has been paid to the study of the activity of doxorubicin complexes with DNA. It has been shown that complexes with Fe^3+^ and Cu^2+^ ions facilitate the binding of doxorubicin to DNA. In addition, they increase the generation of reactive oxygen species, which enhances their cytotoxic effects [11,17,35]. The results of literature studies show that the complexation of doxorubicin with metal ions (Mn^2+^) increases the accumulation of drug in liposomes [36,37].

Furthermore not all of the anthracyclines are able to bind to the selected metal ions. In the case of aclarubicin it is not possible to complex with Fe(III) or Pd(II) ions due to the absence of the phenol groups at the C-11′ position of the aglycone ring [18]. Various ways of metal binding is also apparent from its intrinsic properties. For instance Cu(II): carminomycin complex is formed in a molar ratio of 1:2, while in the case of aclacinomycins: Cu(II): ACLA complexes may occur in two molar ratios: 1:2 and 1:1. In both described complexes an antiferromagnetic connection between the copper ions, even in aqueous solutions, exists [38]. Studies on the interactions of various metal ions essential for living organisms with anthracycline antibiotics allow for a better understanding of the drugs in vivo mechanisms of action.

Metal ions are extremely important in many metabolic processes at the cellular level and the level of the whole organism. One of the important microelements is iron, which is involved in redox reactions, that are associated with the formation of free radicals in the presence of the dioxygen and cause damage to the cell structures. Anthracyclines bind iron to form a drug-metal complexes with a molar ratio of 1:1, 1:2 or 1:3. DOX is able to directly attach iron, and additionally, in the presence of oxygen affects the state of iron oxidation (Fe(II)–Fe(III) cycle). It has been shown that the Fe^3+^ ion can bind three anthracycline molecules in aqueous solution, wherein the metal is chelated by the 11,12-β-ketophenol group [13]. Our results revealed that bivalent metals at a given pH condition can complex metal: DOX at a ratio 2:1 while iron with DOX complexes in a 3:1 ratio.

For the evaluation of cytotoxicity induced by DOX and metals-DOX complexes Apo-Tox GloTM Assay and Luna Logos Biosystems cell counter were used. In our study we observed that metal-DOX complexes are potent inhibitors of MCF-7 cells proliferation. To confirm our results we studied also the influence of all tested metal chlorides in twice lower concentrations than metal-DOX complexes on MCF-7 cell line. Obtained results indicate that simple metal salts don’t exhibit any cytotoxic properties in studied breast cancer cells, therefore we conclude that only the connection of metal with DOX is effective in decreasing of MCF-7 cell viability. We also noticed that the decrease in cell viability was in accordance with reduced cell proliferation. Obtained results indicate that MCF-7 cell viability was clearly decreased in a dose-dependent manner. By the use of the direct count method we observed the most significant decreases in cell viability after 24 h treatment especially with Mg-DOX, Mn-DOX and Ni-DOX complexes in 0.5 µM concentration. The study performed with the use of ApoTox GloTM Assay after 24-h treatment confirmed the obtained results. 2 h and 4 h treatment didn’t change significantly cell number or viability. This is consistent with the results of previous studies of Lange et al., demonstrating unique properties of transition metal-based complexes against cancer cells. Excellent anti-proliferative properties exhibit complexes of transition metals, e.g., iron or copper. An example might be here ferrocifenes that manifest anticancer activity against hormone- dependent and hormone-independent breast cancers [39,40,41]. Copper complexes also show anticancer activity which results from their ability to produce reactive oxygen species. According to the literature the highest efficacy reveals copper complexes, where pyridine-type ligands (pyridine, bipyridine, phenanthroline, etc.) are present or such where copper(I) ion is coordinated to phosphine ligands [42]. In our research copper−DOX complexes weren’t as efficient in cell proliferation inhibition as magnesium or nickel compounds. However, what is important, the effectiveness of metal−DOX complexes against MCF-7 cells hasn’t been under investigation yet. Zinc and copper were also in our investigation of complexes with DOX. According to Milacic et al. these two metals in complexes with pyrrolidine diothiocarbamate showed a significantly high potency in inhibiting the 26S proteasome in intact breast cancer cells [43]. We didn’t observe a significant changes in cell viability, proliferation and apoptosis under the influence of zinc and copper complexes with DOX as compared to free DOX.

The induction of apoptosis in susceptible target cell occurs via the initiation of the death signaling pathways by the cytotoxic agents, such as DOX. Simultaneous or consequent activation of death receptor systems, alterations in mitochondrial function and proteolytic processing of caspases are associated with the activation of apoptosis process by the chemotherapeutic agents [44]. Our results point to the fact that tested compounds, except for zinc and copper−DOX complexes, have caused a significant apoptosis evaluated by caspase 3/7 protein expression in ApoTox Glo^TM^ Assay, which was confirmed for three of the most significant results (Mg-DOX, Mn-DOX and Ni-DOX) by flow cytometry. However, the activity of caspase 3 wasn’t detected in MCF-7 cell line because they do not express detectable levels of caspase-3. Despite the fact that MCF-7 lack the expression of executor caspase 3 known as CPP32, Yama, or apopain, they undergo apoptosis after treatment with anticancer agents. The studied cell line has confirmed 47 kb deletion in exone 3 of the *CPP32* gene. However, it appears that not only the activation of caspase 3, but also caspase 7 by the action of initiator caspases 8 and 9, enhances the apoptosis process. Caspase 7 is strongly associated with caspase 3 and they exhibit the same in vitro substrate specificity [45]. According to McGee et al caspase-3-independant apoptosis could be activated by other effector caspases and then they may overtake the function of caspase-3 in apoptosis enhancement in MCF-7 cells [46]. Our results are in accordance with literature data describing the influence of DOX on executioner caspases activation. Cuvillier et al. showed that the treatment of human breast carcinoma MCF-7 cells with DOX induces apoptosis through cytochrome c release from the mitochondria and activation of the executioner caspase-7 in MCF7 cells which do not express caspase-3 [47]. Additionally we found out that DOX complexed with selected metals is more effective in apoptosis induction than a parent drug. It has been reported that treatment with the other post-transition metal—gallium, which according to its electric charge, ion diameter and electronic configuration is similar to that of Fe^3+^ (transition metal), results in cytochrome c release from the mitochondria, activation of caspase-3 and morphologic changes of apoptosis in human lymphoma cells [48,49]. Therefore we conclude that MCF-7 cell underwent apoptosis under the influence of selected metals-DOX complexes in 0.5 µM concentration after 24 h treatment through the activation of the executioner caspase-7, despite the lack of caspase-3. A constantly emerging topic in anticancer drug research is the development of new compounds that target the cell-cycle checkpoints, which are responsible for the control of the cell-cycle phase progression. For example defects in the G2/M arrest checkpoint may permit a damaged cell to enter mitosis and undergo apoptosis. All of the studies on increasing this effect may enhance cytotoxicity and effectiveness of chemotherapy. The progression of the cell cycle which includes all phases: G1 phase, S phase (DNA replication), G2 phase, and M phase (mitosis and cytokinesis) is essential for cell growth and development. For controlling unlimited growth and cells proliferation, two check points at G1 and G2 phases are important. In cancer cells G2 phase is often intact and left to be critical in cell survival [50]. In order to monitor the connection between cell-cycle arrest, apoptosis and the anti-proliferative effect of metal-DOX complexes in MCF-7 flow cytometry was used. Obtained results indicate that the tested metal-DOX complexes affected the cell cycle. After 24 h treatment with Mn-DOX, Mg-DOX and Ni-DOX in 0.5 µM concentration there was a significant increase in the G2/M phase in comparison to the control and to the DOX-treated cells. We conclude that above described growth and proliferation inhibitory effect of 0.5 µM Mg-DOX, Mn-DOX and Ni-DOX on MCF-7 breast cancer cells is associated with a G2/M arrest in cell cycle progression and with induction of apoptosis. One of the possible mechanisms that explains this phenomenon is the fact that metal-DOX complexes are capable of enhancing cytotoxicity in association with enhanced checkpoints arrest. In the report by Tyagi et al., DOX in combination with silibinin, a derivative of milk thistle, induced increased G2/M arrest and modulated G2/M cell cycle regulators [51]. The results obtained by Tyagi et al. point to the fact that down-regulation of the G2/M cell cycle regulators and G2/M arrest could be a possible mechanism for the effect of DOX on cell growth and apoptosis. A similar mechanism could be responsible for the effect that we observed under the influence of selected transition metals complexed with DOX. To confirm the thesis concerning the importance of cell cycle arrest to DOX cytotoxicity, Ling et al. showed that P388 cells synchronized in S and G2-M phases were characterized by higher sensitivity to DOX than cells in G1 phase [52]. The other literature data also indicate that DOX induces dose-dependent G2/M arrest and that cells in this phase are particularly sensitive to chemical drugs [53].

Although many molecular and cell effects of DOX have been described, including cytotoxicity, apoptosis induction, cell cycle analysis, the ability of selected metals-DOX complexes, e.g., Mg-DOX, Mn-DOX and Ni-DOX complexes to decrease cancer cell viability and to significantly induce apoptosis in MCF-7 cells was demonstrated for the first time. The obtained results led us to the conclusion that DOX-metal complexes may be considered as a potential, new anticancer agents with higher efficacy than free DOX. However further research concerning metal-DOX complexes molecular mechanisms of action and possible changes in oxidative stress signaling pathways under the influence of tested compound are needed.

## 4. Materials and Methods

### 4.1. Reagents

Dulbecco’s modified Eagle’s medium (DMEM), containing glucose at 4.5 mg/mL (25 mM) with Glutamax, penicillin, streptomycin, trypsin–EDTA, FBS Gold and PBS (without Ca and Mg) were provided by Gibco (San Diego, CA, USA). Trypan Blue dye was provided by Sigma-Aldrich (Saint Louis, MO, USA). ApoTox-Glo™ Triplex Assay was provided by Promega (Madison, WI, USA), fluorescein isothiocyanate (FITC) Annexin V Apoptosis Detection Kit I was by BD Pharmingen (San Diego, CA, USA). DOX hydrochloride was obtained from Sigma-Aldrich. Metal chlorides: CuCl_2_∙2H_2_O, ZnCl_2_, MnCl_2_·4H_2_O, MgCl_2_·6H_2_O, NiCl_2_·2H_2_O, CoCl_2_·6H_2_O, FeCl_3_∙4H_2_O, and Tris-HCl buffer were provided by Sigma-Aldrich.

### 4.2. Complexes Preparation

Doxorubicin (0.02 mM) was weighed and dissolved in 10 mL water. NaOH was added to the solution to provide a slightly alkaline pH. Thereafter, the stoichiometric amount of aqueous solution of metal chloride was continuously added under stirring for one hour. The reaction mixture was then stirred for 72 h at room temperature. The obtained solution was incubated for several days to precipitate the sediment complexes. After precipitation, the complexes were filtered with water and dried under vacuum. Elemental composition of the complexes was determined. The obtained complexes had (C_27_H_27_NO_11_)_2_M composition (anhydrous complexes), where M = Cu, Mg and Ni: for the copper complex: %C = 56.51/56.83 (calc/exp) and %H = 4.71/4.59; magnesium complex: %C = 58.57/59.18 (calc/exp) and %H = 4.88/5.06; nickel complex: %C = 56.81/56.53 (calc/exp) and %H = 4.73/4.43; (C_27_H_27_NO_11_)_2_M·0.5H_2_O composition for cobalt complex: %C = 56.35/56.33 (calc/exp) and %H = 4.78/4.49; (C_27_H_27_NO_11_)_3_Fe·0.5H_2_O composition for the iron complex: %C = 57.58/57.42 (calc/exp) and% H = 4.85/4.61 (calc/exp) and (C_27_H_27_NO_11_)_2_M·H_2_O, where M = Zn, Mn: for the zinc complex: %C = 55.6/55.39 (calc/exp) and %H = 4.81/4.42; and manganese complex: %C = 56.10/56.23 (calc/exp) and %H = 4.84/4.91. IR spectra were also recorded. The obtained complexes are water–soluble.

### 4.3. Infrared Spectrum (FT-IR, ATR) and Raman Spectrum of DOX and Metal Complexes

The FT-IR spectra for DOX hydrochloride were recorded with an Alfa spectrometer (Bruker, Billerica, MA, USA) within the range of 400–4000 cm^−1^. Samples in the solid state were measured in KBr matrix pellets and ATR technique. FT-Raman spectra of solid samples were recorded in the range of 400–4000 cm^−1^ with a MultiRam (Bruker) spectrometer. Experimental spectra were interpreted in terms of literature data [54]. IR spectra (KBr) metal complexes were also registered to determine metal-ligand coordination mode.

### 4.4. Study on the Composition of Metal-DOX Complexes in Aqueous Solutions

Aqueous solutions of CuCl_2_, FeCl_3_, ZnCl_2_, MgCl_2_, MnCl_2_, CoCl_2_ and NiCl_2_ at concentrations of 0.01 mol/L were prepared for the study of complex composition in the aqueous solutions. DOX solution (50 μmol/L) was prepared in TRIS-HCl buffer at 0.02 mol/L and pH 7.01. Composition of DOX complexes with metals was determined by spectrophotometric titration. Three mL of DOX solution in buffer was poured into the cuvette (quartz) and the absorbance of the solution against the reference TRIS-HCl buffer was measured. A solution of metal chloride at a concentration of 0.01 mol/L was then added in the amounts shown in Table 3.

After each addition, the solution was stirred in the cuvette and the absorbance of the solution was measured. DOX was complexed with iron, copper, cobalt, manganese, nickel, magnesium and zinc at pH 7.01. For complexes with fixed composition in solution, the measurement was repeated after 24 h to determine the stability of the complexes. UV-VIS spectra were recorded in the range of 190–800 nm with a HACH 2000 spectrometer (HACH Company, Loveland, CO, USA).

### 4.5. Cell Culture

The effect of DOX, Cu-DOX, Zn-DOX, Co-DOX, Ni-DOX, Fe-DOX, Mn-DOX and Mg-DOX was examined in MCF-7 breast cancer cell line, which were obtained from the American Type Culture Collection (ATCC, University Boulevard, Manassas, VA, USA). Cells were maintained in DMEM supplemented with 10% FBS, penicillin (100 U/mL), and streptomycin (100 μg/mL) at 37 °C in a humified atmosphere of 5% CO_2_ in air. Adherent cells (2 × 10^5^ cells/mL) in 2 mL of culture medium were incubated with the test compounds in tissue culture 6-well plates. MCF-7 cells (2 × 10^4^ cells/mL) in 200 µL of culture medium were incubated without and with the test compounds in tissue culture treated black 96-well plates for the ApoTox-Glo Assay. The cells proliferation, viability, cytotoxicity and apoptosis were estimated at Dox, Cu-DOX, Zn-DOX, Co-DOX, Ni-DOX, Fe-DOX, Mn-DOX and Mg-DOX concentration of 0.5 µM, 0.1 µM, 0.05 µM and 0.01 µM. Detection of apoptosis and necrosis and cell cycle analysis were conducted with DOX and selected metal-DOX complexes: Ni-DOX, Mn-DOX and Mg-DOX concentration of 0.5 µM.

### 4.6. Cell Exposure to DOX and Its Metal Complexes

DOX, Cu-DOX, Zn-DOX, Co-DOX, Ni-DOX, Fe-DOX, Mn-DOX and Mg-DOX were stored in a refrigerator at temperature 4 °C. The compounds were added to the cultured cells for a final concentration in the range of 0.01 µM to 0.5 µM. Concentration range was selected for the experiments on the basis of previously established data. According to the literature data at DOX concentrations of 0.05 µM, 0.1 µM and 0.5 µM MCF-7 cells growth is inhibited by approximately 15, 50, and 75%, respectively [55]. The control cells were incubated for 24 h with and without the test compounds for: the ApoTox-Glo^TM^ assay, the estimation of the cells proliferation by using cell counter, cell cycle analysis and apoptosis and necrosis detection.

### 4.7. Estimation of Cells Proliferation

The number MCF-7 cells with division into living and dead after 2 h, 4 h and 24 h treatment, was determined by direct counts of cells with the use of trypan blue dye using a LUNA Logos Biosystems cell counter.

### 4.8. Estimation of Metals Cytotoxicity

To measure metal chlorides cytotoxicity CytoTox-Glo Cytotoxicity Assay (Promega Corporation, Madison, WI, USA) was used. The measurement was conducted according to manufacturer’s protocol. In brief, assay uses a luminogenic peptide substrate (alanyl-alanyl-phenylalanyl-aminoluciferin; AAF-Glo™ Substrate) to measure “dead-cell protease activity”, which is released from cells that have lost membrane integrity. Substrate cannot cross the intact membrane of live cells, therefore the assay detects dead cells and relies on the properties of a proprietary thermostable luciferase. Applied luciferase uses aminoluciferin as a substrate to generate a stable “glow-type” luminescent signal. Luminescence was measured with a GloMax^®^-Multi Microplate Multimode Reader plate reader (Promega Corporation, Madison, WI, USA).

### 4.9. Estimation of Cells Viability, Cytotoxicity and Apoptosis

To measure MCF-7 cells viability, cytotoxicity and apoptosis, the ApoTox-Glo™ Triplex Assay (Promega) was used. All the measurements were conducted on the same sample according to manufacturer’s protocol. In brief, in the first part two protease activities were measured simultaneously as a markers of cells viability and cytotoxicity. After adding an appropriate substrate: GF-AFC (for viability) and bis-AAF-R110 (for cytotoxicity), live-cell or dead-cell proteases cleave added compound and fluorescent signal is produced. Apoptosis was measured after the addition of a luminogenic caspase 3/7 substrate (Caspase Glo 3/7), which is subsequently cleaved in apoptotic cells to produce a luminescent signal. Fluorescence at 365 Ex/500 Em (viability), 485 Ex/535 Em (cytotoxicity) and luminescence (apoptosis) were measured with a GloMax^®^-Multi Microplate Multimode Reader plate reader.

### 4.10. Detection of Apoptosis and Necrosis

The MCF-7 cells were exposed to DOX and Ni-DOX, Mn-DOX and Mg-DOX in the high glucose DMEM for 24 h. Apoptosis and necrosis were evaluated by flow cytometry on FACSCanto II cytometer (Becton-Dickinson). The cells were trypsinised and resuspended in DMEM. After that time, the cells were suspended in binding buffer for staining with FITC-Annexin V and propidium iodide—PI for 15 min at room temperature in the dark following the manufacturer’s instructions (FITC Annexin V apoptosis detection Kit I). The signal obtained from cells stained with annexin V or PI alone was used for fluorescence compensation. Data were analyzed with FACSDiva software.

### 4.11. Cell Cycle Assay

The MCF-7 cells were exposed to DOX and Ni-DOX, Mn-DOX and Mg-DOX in the high glucose DMEM for 24 h. Subsequently the cells were trypsinized and resuspended in cold PBS. Cells (1 × 10^6^) were collected by centrifugation at 1000 rpm for 5 min. The cell pellets were washed twice with cold PBS and resuspended approximately with 100 µL of cold PBS. The cell pellets were fixed with ice cold 70% ethanol for minimum 1 h at 4 °C. The fixed cells were centrifuged at 1200 rpm for 5 min. Following, the cell pellets were washed twice with cold PBS and resuspended in the 38 mM sodium citrate, containing 120 µg/mL RNAse A and 10 µg/mL propidium iodide at 37 °C for 30 min in darkness. Then cells were analyzed for determination of cell cycle phases using FACSCanto II cytometer (Becton-Dickinson).

### 4.12. Statistical Analysis

For parametric data one-way analysis of variance (ANOVA) followed by a Tukey test was applied. Results from five independent experiments were expressed as mean ± standard deviation (SD) of mean for parametric data. Significance was considered when *p* ≤ 0.05. Statistica 13.0 (StatSoft, Kraków, Poland) was used.

## Figures and Tables

**Table 1:** Possible points of metal attachment to the anthracycline [13,15,16,17,18,19].

Metal Ion	Possible Points of Anthracycline Attachment
Fe(II)	Carbonyl group (at C-12′)
	Phenolic group (at C-11′)
Cu(II)	Carbonyl group (at C-5′)
	Phenolic group (at C-6′)
	Carbonyl group (at C-12′)
	Phenolic group (at C-11′)
Yb(II)	Carbonyl group (at C-12′)
	Phenolic group (at C-11′)

**Table 2:** Wavenumbers (cm^−1^), intensities and assignments of bands occurring in the IR (KBr, ATR) and Raman spectra of DOX hydrochloride and IR spectra for Cu-DOX complex.

Doxorubicin			Cu Complex	Assingments (Numbering Atoms in Figure 5)
IR KBr	IR ATR	Raman	IR KBr	
3423 vs			3434 vs	νHO-H
3026 w		3047 w		
2932 m		2940 vs	2922 m	νC(33,35,37)-H 33/35/37 H
2899 m		2898 s	2854w	νC(33,35)-H
1729 m	1731 w	1732 w	1724 w	δN-H
		1643 s		νRing, δCO(25)-H
1617 m	1615 m	1607 m	1622 m	ring
1583 m	1583 m	1581 m	1568 m	ring
1524 w	1524 w		1515 w	sδC(20,22)-H_2_, sδCO(11,25)-H 20/22
1462 m	1463 m	1461 s	1443 m	Ring, δC(30)-H
		1428 s		Ring-O, Ring=O, δC(22,20,15)-H_2_, δC(13,31,33,37)-H, δC(32)-H_2_
1413 m	1413 m	1404 m	1415 m	Ring, C-H_x_(ar)
1379 m		1374 w		δC(13)-H, δO-H^…^O, δC-C, C(16)-OH
		1329 w		δO-H^…^O, Ring, C(20,15)-H_2_, δC(16)-OH, C(13)-H
1285 s	1286 vs	1290 m	1279 m	δO-H^…^O, Ring, ouC(32)-H_2_, δC(11)-OH, C(13)-H
1236 m	1236 m	1247 m		C(32)-H_2_, C(33,35,37)-H, O(36)-H, C-O(38)-C
1211 m	1209 m	1205 s	1211 m	δO-H^…^O, Ring, ouC(20)-H_2_, δC(11)-OH, N-H_2_
1153 w	1149 vw	1158 vw	1150 w	Ring external, C(33)-NH^+^, HC(37)-C(35)H, C(39)-H_3_
1116 m	1116 m	1119 vw	1116 m	Ring breathing, δC(16)-C(18)=O, C(30)-H_3_,
1074 m	1074 m	1085 w	1075 m	δC(16)-C(15)-H, C(4)-O(6)-C(30), δN-H_2_, δC-Hx (ali)
1007 m		1009 vw	1013 m	νC(13)-O(14)-C(31), νC(16)-O(17)H, δC-Hx (ali)
991 m	997 vs	989 vw	986 m	C(20)-H_2_ (ali), δC=O, δC(16)-OH, δC(16)C(18)-C(22)
970 m	970 m			δC-C(27)=O, C(24)-OH, δC-Hx (ali)
950 w	945 w			δC-Hx, CO(14)-C(31), O-C(31)-O
912 vw	913 w	915 vw		δC(24)-OH, δC(22)-H_2_, O(28)^…^H-O(25)
804 w	804 m	795 w	809 w	ouRing
762 w	765 w	764 vw	765 w	δRing(C-H), ωC(31,35)-H, ωC(39)-H_3_,

vs, s, w, vw, m. ou, ali and aro represent very strong, strong, weak, very weak, medium high band intensity, out-of-plane vibration, aliphatic and aromatic group; ν, δ, ω belong to the stretching, bending, and wagging vibration; C_x_-H_y_ represents xth carbon with y number of hydrogen.

**Table 3:** Quantities of DOX and metal chloride.

Sample Number	Molar Ratio Metal:DOX	DOX (mL)	Metal Chloride (μL)
1	0	3	0
2	0.13	3	2
3	0.2	3	3
4	0.3	3	4
5	0.33	3	5
6	0.5	3	7.5
7	1	3	15
8	1.25	3	20
9	2	3	30
10	3.25	3	50

## Abbreviations

**DOX**	Doxorubicin
Cu-DOX	Complex of doxorubicin with Cu (molar ratio 1:2)
Zn-DOX	Complex of doxorubicin with Zn (molar ratio 1:2)
Co-DOX	Complex of doxorubicin with Co (molar ratio 1:2)
Ni-DOX	Complex of doxorubicin with Ni (molar ratio 1:2)
Fe-DOX	Complex of doxorubicin with Fe (molar ratio 1:3)
Mn-DOX	Complex of doxorubicin with Mn (molar ratio 1:2)
Mg-DOX	Complex of doxorubicin with Mg (molar ratio 1:2)
